# De novo biosynthesis of berberine and halogenated benzylisoquinoline alkaloids in *Saccharomyces cerevisiae*

**DOI:** 10.1038/s42004-023-00821-9

**Published:** 2023-02-09

**Authors:** Jianing Han, Sijin Li

**Affiliations:** Robert F. Smith School of Chemical and Biomolecular Engineering, Cornell University, Ithaca, NY 14853 USA

**Keywords:** Synthetic biology, Metabolic pathways, Natural product synthesis, Multienzyme complexes

## Abstract

Berberine is an extensively used pharmaceutical benzylisoquinoline alkaloid (BIA) derived from plants. Microbial manufacturing has emerged as a promising approach to source valuable BIAs. Here, we demonstrated the complete biosynthesis of berberine in *Saccharomyces cerevisiae* by engineering 19 genes including 12 heterologous genes from plants and bacteria. Overexpressing bottleneck enzymes, fermentation scale-up, and heating treatment after fermentation increased berberine titer by 643-fold to 1.08 mg L-1. This pathway also showed high efficiency to incorporate halogenated tyrosine for the synthesis of unnatural BIA derivatives that have higher therapeutical potentials. We firstly demonstrate the in vivo biosynthesis of 11-fluoro-tetrahydrocolumbamine via nine enzymatic reactions. The efficiency and promiscuity of our pathway also allow for the simultaneous incorporation of two fluorine-substituted tyrosine derivatives to 8, 3’-di-fluoro-coclaurine. This work highlights the potential of yeast as a versatile microbial biosynthetic platform to strengthen current pharmaceutical supply chain and to advance drug development.

## Abstract (web summary)

Berberine is a plant-derived benzylisoquinoline alkaloid with diverse pharmaceutical activities, however, the production of berberine from medicinal plants and organic synthesis remains unsustainable. Here, the authors report the complete biosynthesis of berberine in yeast by engineering genes from plants and bacteria.

## Introduction

Berberine is a plant-derived benzylisoquinoline alkaloid (BIA) with multiple pharmaceutical activities. It exerts wide-spectrum antimicrobial and antiviral effects against pathogenic bacteria such as *Mycobacterium tuberculosis* and *Staphylococcus aureus*^1^, and viruses such as influenza and herpes^2–4^. Recently, berberine has been found with pharmaceutical potential to treat obesity^5^, regulate gut microbiota, treat atherosclerosis^6^, or ameliorate Parkinson’s disease^7^. There have been over 80 clinical trials worldwide to investigate berberine’s medicinal uses in these diseases, along with its anticancer, cardioprotective, and anti-inflammatory potentials^1,8^.

Berberine production largely relies on extraction and isolation from medicinal plants. A variety of medicinal plants such as goldenseal (*Hydrastis canadensis*), barberry fruit (*Berberis vulgaris*), and goldthreads (*Coptis genus*) can synthesize berberine as an important specialized metabolite. These medicinal plants have been widely used in Ayurvedic, Chinese, and Middle Eastern traditional medicines^9^ to treat diseases such as wound infections and diarrhea. However, extraction from plants^9^ relies solely on time-consuming agricultural processes and is susceptible to environmental changes. Meanwhile, the chemical synthetic process of berberine is not environmental-friendly and remains challenging due to the complexity of berberine structure. Although several approaches for the chemical synthesis of berberine were reported^10–12^, the complicated steps and uses of heavy metals hinder future manufacturing.

Microbial manufacturing provides a new strategy to address the challenges of plant or chemical approaches. Fermentation in a tractable microbial host such as *Saccharomyces cerevisiae* (baker’s yeast) is more rapid, cost-effective, efficient, and environmental-friendly^13–15^. Yeast has proven a powerful platform to produce valuable and complex plant natural products (PNPs), such as artemisinin^16^, cannabinoids^17^, scopolamine^18^, and vinblastine^19^. As the model eukaryotic microorganism, yeast provides the endomembrane system for the functional expression of plant membrane-bound enzymes, such as cytochrome P450s^20^. The well-studied yeast endogenous metabolism and advanced genetic tools enable the reconstruction and engineering of heterologous pathways in yeast^21^. Moreover, the in vivo biosynthetic process in yeast makes it feasible to produce tailor-made PNP derivatives with higher bioactivity and bioavailability. Enzymatic transformation of modified small-molecule substrates in yeast allows for the synthesis of unnatural PNPs; adding tailoring enzymes such as halogenases to the established PNP biosynthetic pathway allows for novel structural modifications based on the PNP scaffold^15^. These strategies have led to the synthesis of several halogenated pharmaceutical BIAs^22^ and monoterpene indole alkaloids (MIAs)^23–25^. The strategies are uniquely suitable for improving the pharmacokinetic properties of berberine and other BIAs. Despite the wide therapeutic uses of berberine, the membrane permeability hinder the oral bioavailability of berberine to below 5%^26^. Halogenation is an important strategy in drug discovery and development to increase a PNP’s bioavailability and bioactivity^27^. In particular, fluorination is uniquely important in medicinal chemistry, as the fluorine substitution can increase the lead compound’s binding efficacy, and selectivity significantly^28^. It can be anticipated that manufacturing the halogenated derivatives of BIAs in yeast will significantly contribute to drug development and discovery. Several halogenated BIAs with a classical benzylisoquinoline structure have been synthesized using in vivo or in vitro approaches. In vitro enzymatic approaches were reported to produce various derivatives of norcoclaurine and coclaurine^29–31^. The in vitro synthesis of halogenated BIAs downstream of these two chemicals have not been achieved, possibly due to the difficulty in expressing the essential membrane-bound cytochrome P450 enzymes. The in vivo approach using established BIA biosynthetic pathways allows for the synthesis of more complicated BIAs such as reticuline^22^, yet it remains challenging to biosynthesize more complex structures, such as halogenated protoberberines.

The biosynthetic pathway of berberine has been partially elucidated and reconstructed in yeast, with a berberine titer of 39 µg L^−1^ from the substrate norlaudanosoline^32^. However, the limited titer and the use of substrate norlaudanosoline impeded further industrial applications. With the development of plant genomics, bioinformatics, and synthetic biology, the de novo biosynthetic pathways of BIAs^33^ that share similar synthetic routes with berberine, such as reticuline, noscapine and sanguinarine, have been identified using their native producers^34–36^ and reconstructed in yeast^22,33,37–39^. These pioneering studies proved the feasibility to engineer a de novo and efficient berberine-producing strain.

In this study, we reconstructed a complete biosynthetic pathway of berberine in yeast. The initial strain was engineered with 15 enzymes, including 12 heterologous enzymes from plants and bacteria, and can produce 1.68 µg L^−1^ berberine from glucose. Engineering of rate-limiting enzymes improved the berberine titer by 20-fold to 35.1 µg L^−1^. Batch fermentation in a 0.75-L bioreactor followed by a heating treatment to help the conversion, the berberine titer was finally improved to 1.08 mg L^−1^, 643-fold of the initial production. We also demonstrated the production of several novel halogenated BIAs by feeding halogenated tyrosines, including 3-fluoro-tyrosine, and 3-chloro-tyrosine, and 3-iodo-tyrosine, in the optimized berberine-producing strain. The engineered yeast showed high compatibility to incorporate fluorine-substituted tyrosine derivatives, which led to the production of a fluorine-substituted tetrahydrocolumbamine via nine enzymatic transformations, which is the first time to synthesize halogenated protoberberines in vivo. In addition, the engineered BIA biosynthetic pathway allows for the condensation and modification of two fluorine-substituted tyrosine derivatives towards a difluoro BIA product. The de novo berberine-producing yeast strains and fermentation strategy developed in this study will lead to an economic, controllable, and robust supply chain of berberine, as well as an efficient yeast platform to synthesize halogenated BIAs that used to be inaccessible for future drug discovery and development.

## Results

### Construction of a de novo berberine biosynthetic pathway in yeast

**Fig. 1:**
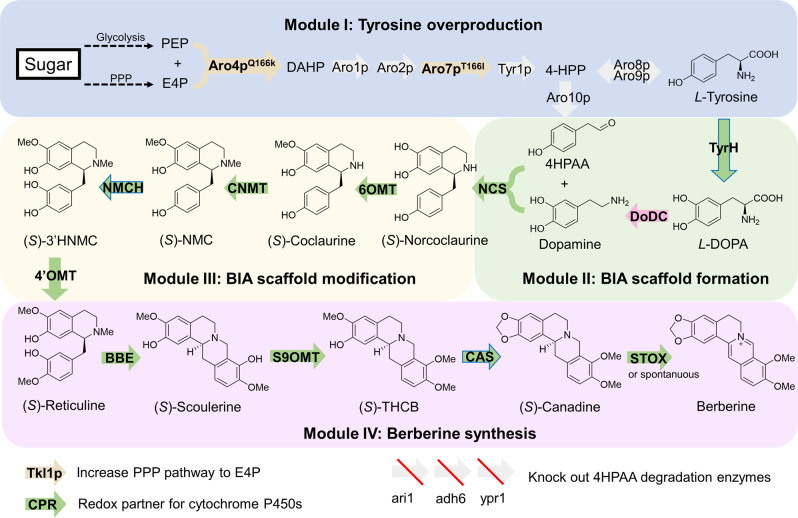
Design of biosynthetic pathways for de novo production of berberine in *S. cerevisiae*. Block arrows indicate enzyme-catalyzed reactions. Green arrows, plant enzymes; pink arrows, bacteria enzymes; yellow arrows, overexpressed and modified yeast enzymes; gray arrows, unmodified yeast enzymes. Blue borders of arrows indicate cytochrome P450 enzymes. Arrows with red slashes indicate knocked out enzymes. Enzyme abbreviations: Aro10p, phenylpyruvate decarboxylase; Aro1p, pentafunctional arom enzyme; Aro2p, bifunctional chorismate synthase and flavin reductase; Aro4p^Q166K^, mutated phospho-2-dehydro-3-deoxyheptonate aldolase with relieved tyrosine-inhibition; Aro7p^T226I^, mutated chorismate mutase with relieved tyrosine-inhibition; Aro8p, aromatic aminotransferase I; Aro9p, aromatic aminotransferase II; BBE, berberine bridge enzyme; CAS, canadine synthase; CNMT, coclaurine *N*-methyltransferase; CPR, cytochrome P450 reductase; DODC, *L*-DOPA decarboxylase; NCS norcoclaurine synthase, NMCH *N*-methylcoclaurine hydroxylase, STOX tetrahydroprotoberberine oxidase, S9OMT scoulerine 9-*O*-methyltransferase, Tkl1p transketolase, Tyr1p prephenate dehydrogenase, TyrH tyrosine hydroxylase, 4′OMT, 3′-hydroxy-*N*-methylcoclaurine 4′-*O*-methyltransferase; and 6OMT norcoclaurine 6-*O*-methyltransferase. Compound abbreviations: DAHP 3-deoxy-*D*-arabino-2-heptulosonic acid 7-phosphate, E4P erythrose 4-phosphate, *L*-DOPA *L*-3,4-dihydroxyphenylalanine, NMC *N*-methylcoclaurine, PEP phosphoenolpyruvate, THCB tetrahydrocolumbamine, 4HPAA 4-hydroxyphenylacetaldehyde; 4-HPP 4-hydroxyphenylpyruvate. PPP pentose phosphate pathway.

**Fig. 2:**
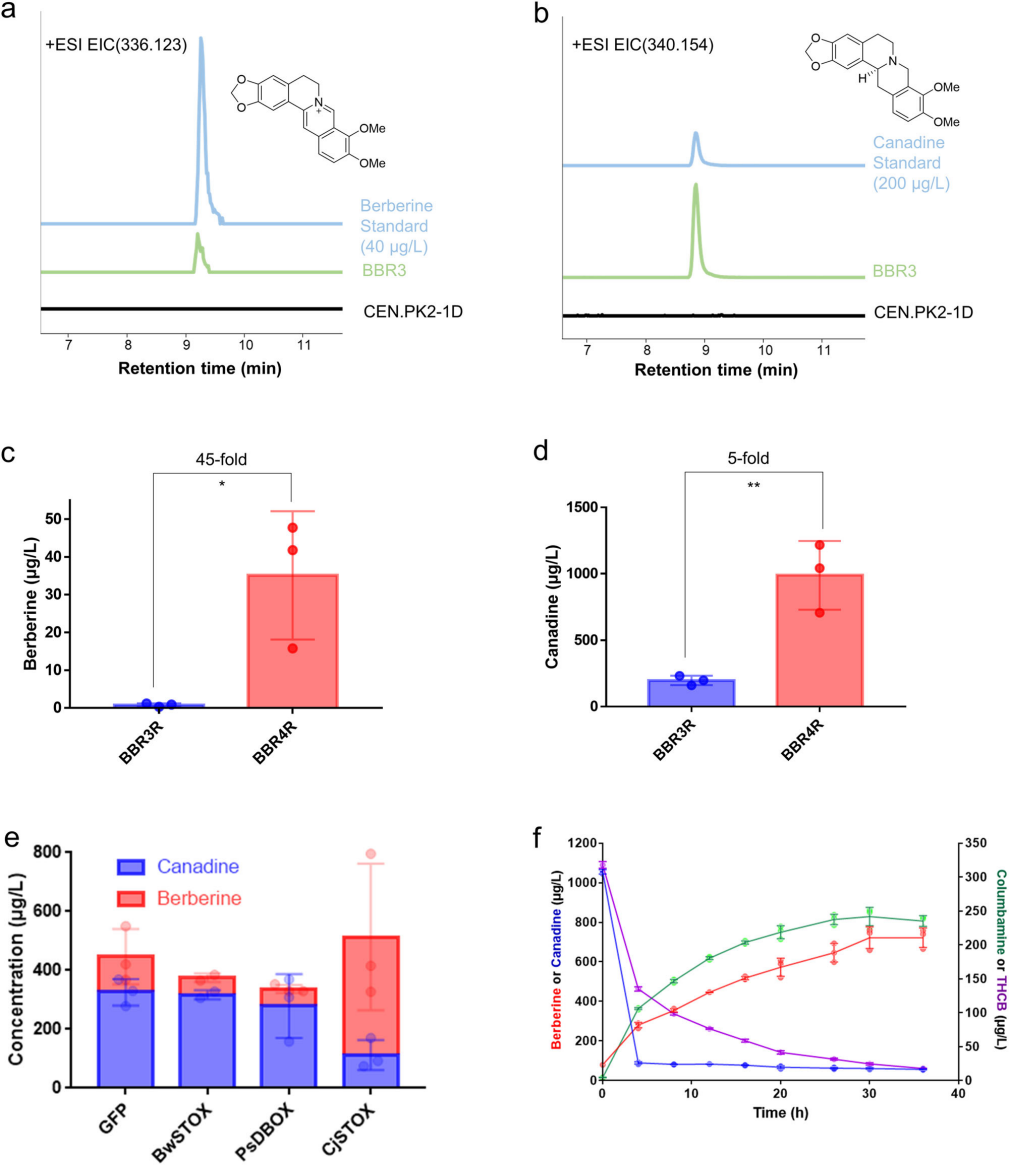
Optimizations of the strain and bioprocess. Production of (**a**) berberine, and (**b**) canadine, in strain BBR3. Extracted ion chromatogram (EIC) of berberine (m/z 336.123) and canadine (m/z 340.154) are shown. Traces are representative of three biological replicates. Titer of (**c**) berberine, and (**d**) canadine, in optimized strain BBR4R compared with original BBR3R. Two-tailed *t*-test was used to calculate the *p*-value: *p < 0.05 and **p < 0.01. **e** Screening of active STOX in *N. benthamiana* transient expression system with canadine infiltrated. **f** Increased concentration of berberine and possible intermediates under heating condition. Independent experiments, *n* = 3. Error bars represent standard deviation.

We constructed the *de novo* berberine biosynthetic pathway with four modules in yeast (Fig. 1) with a total of 12 heterologous enzymes. Module I overproduces *L*-tyrosine by the optimization of the yeast endogenous aromatic acid biosynthetic pathway, which has been constructed in previous work^40^. Yeast endogenous transketolase 1 (TKL1), phospho-2-dehydro-3-deoxyheptonate aldolase variant (ARO4^Q166K^), and chorismite mutase variant (ARO7^T226I^) were overexpressed in this strain with an antibiotic hygromycin resistant marker (HygR), namely ySL14^40^. Module II leads to the formation of the BIA scaffold norcoclaurine, which contains four enzymes including tyrosine hydroxylase (TyrH), cytochrome P450 reductase (CPR), dihydroxyphenylalanine decarboxylase (DoDC), and norcoclaurine synthase (NCS). Both mammalian^22,37,41,42^ and plant^38,43^ TyrH were reported in previous studies. We chose the plant-derived TyrH, namely CYP76AD5 from *Beta vulgaris* (sugar beets), due to its high activity reported in the previous study^43,44^. Together with the TyrH, the cytochrome P450 reductase (CPR) from *Arabidopsis thaliana*, namely ATR1, NCS from *Coptis japonica* with N-terminal 35 amino acids truncated^43^, DoDC from *Pseudomonas putida*, and a His5 auxotrophic marker, were integrated into the YGL157W (*ari1*) site of ySL14, resulting in strain BBR1. The *ari1* gene encodes an aldehyde reductase that leads to the degradation of 4-hydroxyphenylacetic acid (4HPAA)^43^, a precursor of norcoclaurine, and thus was deleted during the integration. *De novo* norcoclaurine production in strain BBR1 was confirmed by high-resolution liquid chromatography coupled with mass spectrometry (LC-MS) (Supplementary Fig. S1a), after culturing the strain in YPD medium at 30 °C for three days and analyzing the supernatant of the culture medium by LC-MS. Module III contains four enzymes that convert norcoclaurine to reticuline, a key intermediate in the BIA pathway. Norcoclaurine 6-*O*-methyltransferase (6OMT), coclaurine *N*-methyltransferase (CNMT), 3’-hydroxy-*N*-methylcoclaurine 4’-*O*-methyltransferase (4’OMT) from *Papaver somniferum* (opium poppy), *N*-methylcoclaurine 3’-hydroxylase (NMCH) from *Eschscholzia californica* (California poppy), and a Leu2 auxotrophic marker, were integrated into the YMR318C (*adh6*, encoding an alcohol dehydrogenase degrading 4HPAA^43^) site of BBR1, resulting in the strain BBR2, with reticuline production verified (Supplementary Fig. S1b). Module IV contains four enzymes that lead to the production of berberine. Berberine bridge enzyme (BBE), scoulerine 9-*O*-methyltransferase (S9OMT) from *P. somniferum*, canadine synthase (CAS) from *C. japonica*, tetrahydroprotoberberine oxidase (STOX) from *Berberis wilsoniae*, and a Ura3 marker were integrated into the YDR368W (*ypr1*, an aldo-keto reductase degrading 4HPAA^43^) site of BBR2. The resultant berberine-producing strain BBR3 contains 19 modified or engineered genes, including 15 genes overexpressed and 4 endogenous gene deleted. This strain produced 217.61 µg L^−1^ canadine and 1.68 µg L^−1^ berberine (Fig. 2a, b), after cultured in YPD medium at 30 °C for three days.

### Pathway flux optimization

As three auxotrophic markers, His5, Leu2, Ura3, and one antibiotic marker HygR have been occupied in BBR3, we rescued the markers for further engineering by Cre-LoxP system^45^. The rescued strain named BBR3R showed the same level of berberine production as BBR3 (1.39 µg L^−1^), indicating the markers did not impact the BIA synthesis in YPD medium. NCS, 4’OMT and CAS were reported to be the bottleneck steps^39,43^. We incorporated one additional copy of the three enzymes each, along with Leu2, to optimize the pathway flux; the new copies were integrated into the YDR541C site, which is another 4HPAA-degrading gene based on a previous study^43^, resulting in BBR4. The Leu2 marker in BBR4 was then rescued, leading to a new strain named BBR4R. The optimized strain BBR4R produced 35.10 µg L^−1^ berberine, 20-fold higher than the original strain BBR3R (Fig. 2c). Canadine production in BBR4R was also increased by 5-fold to 989.10 µg L^−1^ (Fig. 2d).

The high accumulation of canadine and relatively low berberine production indicates that the canadine to berberine conversion catalyzed by STOX is another major bottleneck in the berberine pathway. We first tried to overexpress the enzyme STOX from *B. wilsoniae* (BwSTOX)^46^ using high-copy plasmids, including pAG424 and pAG425^47^, in BBR4R to improve the conversion. However, no berberine accumulation was detected from the strains harboring the STOX-expressing plasmids. As the strains must be cultured in synthetic dropout (SD) medium to maintain the plasmids, the lack of berberine production is likely related to the lower pH or less nutrients in SD medium than in YPD medium. Therefore, we changed the original auxotrophic Leu2 marker on the yeast high-copy plasmid pAG425 with an antibiotic hygromycin B resistant marker, HygR, leading to a new plasmid named pAG42H, which allows for selective growth in YPD medium with hygromycin B. BwSTOX was overexpressed on the pAG42H vector, yet it did not improve the conversion, indicating the BwSTOX is not active in yeast, as postulated in previous study^32^.

To figure out if the inactivity of BwSTOX comes from its incompatibility with the yeast expression system, we validated the enzyme’s activity in the model plant *Nicotiana benthamiana* by transient expression^48^. *Agrobacterium tumefaciens* GV3101 containing BwSTOX was infiltrated to *N. benthamiana* leaves. Meanwhile, we also infiltrated other STOX variants from *C. japonica* (CjSTOX)^49^ and *P. somniferum* (PsDBOX)^50^ to screen for more STOX candidates. After four days for gene expression, canadine was infiltrated as the substrate. Metabolites were then extracted from the leaves 24 hours later and analyzed by LC-MS. Only CjSTOX showed activity in the test, with a higher berberine and lower canadine concentrations (Fig. 2e) compared to the control that has no STOX. However, overexpression of CjSTOX in yeast still did not increase berberine production. Despite of significant trouble shooting efforts, including fusing the CjSTOX with mCherry proteins to confirm the expression (Supplementary Fig. S2a), relocalizing the CjSTOX on ER or vacuole by signal peptide engineering (Supplementary Fig. S2b and S2c, method described in Supplementary Method), and feeding additional high concentration of canadine, we did not observe the activity of CjSTOX to convert canadine to berberine in yeast.

### Chemical oxidation to increase berberine production

Due to the lack of active STOX in yeast system, we postulated that the berberine was produced by spontaneous chemical reaction, which was also discussed in a previous study when norlaudanosoline was used as the feeding substrate^32^. Therefore, we continued to optimize the conversion for higher berberine titer under mild conditions. Temperature was found to be an important parameter in the chemical reaction via a preliminary test. 1 mg L^−1^ canadine dissolved in water was heated at 55 °C or 98 °C for four hours. The consumption of canadine and production of berberine were observed under both conditions and the treatment at 98 °C showed 40.3% conversion, higher than the 1.90% conversion under 55 °C (Supplementary Fig. S3a). Then we heated the culture supernatant of strain BBR4R at 98 °C for 36 h and collected samples at 4, 8, 12, 16, 20, 26, 30, 36 h for LC-MS analysis. There were 1.06 mg L^−1^ canadine and 49.5 µg L^−1^ berberine in the supernatant before treatment. Berberine concentration gradually increased during the treatment while canadine concentration dropped to 88.9 µg L^−1^ in the first four hours and was continuously consumed. After heating, 721 µg L^−1^ berberine was obtained and nearly all canadine was consumed (Fig. 2f). Similar to the oxidation converting canadine to berberine via the removal of 4 H atoms, we found a conversion of tetrahydrocolumbamine (THCB, m/z 342.170) to putative columbamine (m/z 338.139) (Fig. 2f). The possible mechanism and molecular structures are shown in Supplementary Fig. S3b.

### Fermentation scale-up in bioreactors

**Fig. 3:**
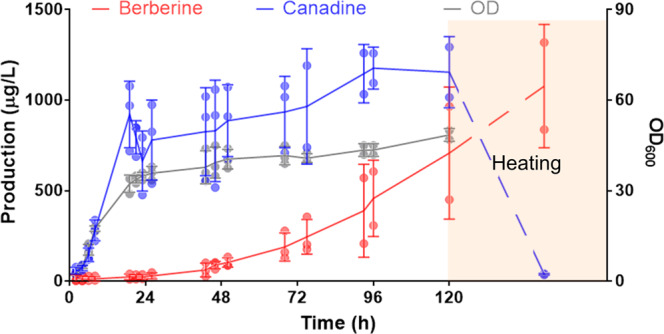
Scale-up fermentation of BBR4R strain. Berberine titer, canadine titer and OD600 during the bioprocessing followed by a 36-hour heating are shown. Error bars represent either standard deviation of three independent fermentations for points before 72 h or of two independent fermentations for points after 72 h.

To further evaluate the scalability and industrial potential of the berberine-producing strain, three batches of 0.75-L scale fermentations were performed in a 2.5-L Eppendorf BioFlo 310 bioreactor. BBR4R was cultivated in YPD at 30 °C for five days. 708.3 µg L^−1^ berberine and 1.15 mg L^−1^ canadine were produced, with a final OD600 of 48.5 (Fig. 3). Compared to the small-scale cultivation, the berberine titer in the bioreactor increased by 20-fold from 35.1 to 708.3 µg L^−1^. We hypothesize that the improved air supply and agitation in the bioreactor increased oxygen transfer and helped the spontaneous chemical oxidation of canadine to berberine. The supernatant was then heated at 98 °C for 36 h and finally achieved 1.08 mg L^−1^ berberine production with all canadine consumed (Fig. 3). Compared with the previously reported berberine production (39 µg L^−1^) from norlaudanosoline in yeast^32^, our work increased the titer by 26.7-fold to 1.08 mg L^−1^ of berberine from glucose in yeast.

### Production of halogenated BIA derivatives

**Fig. 4:**
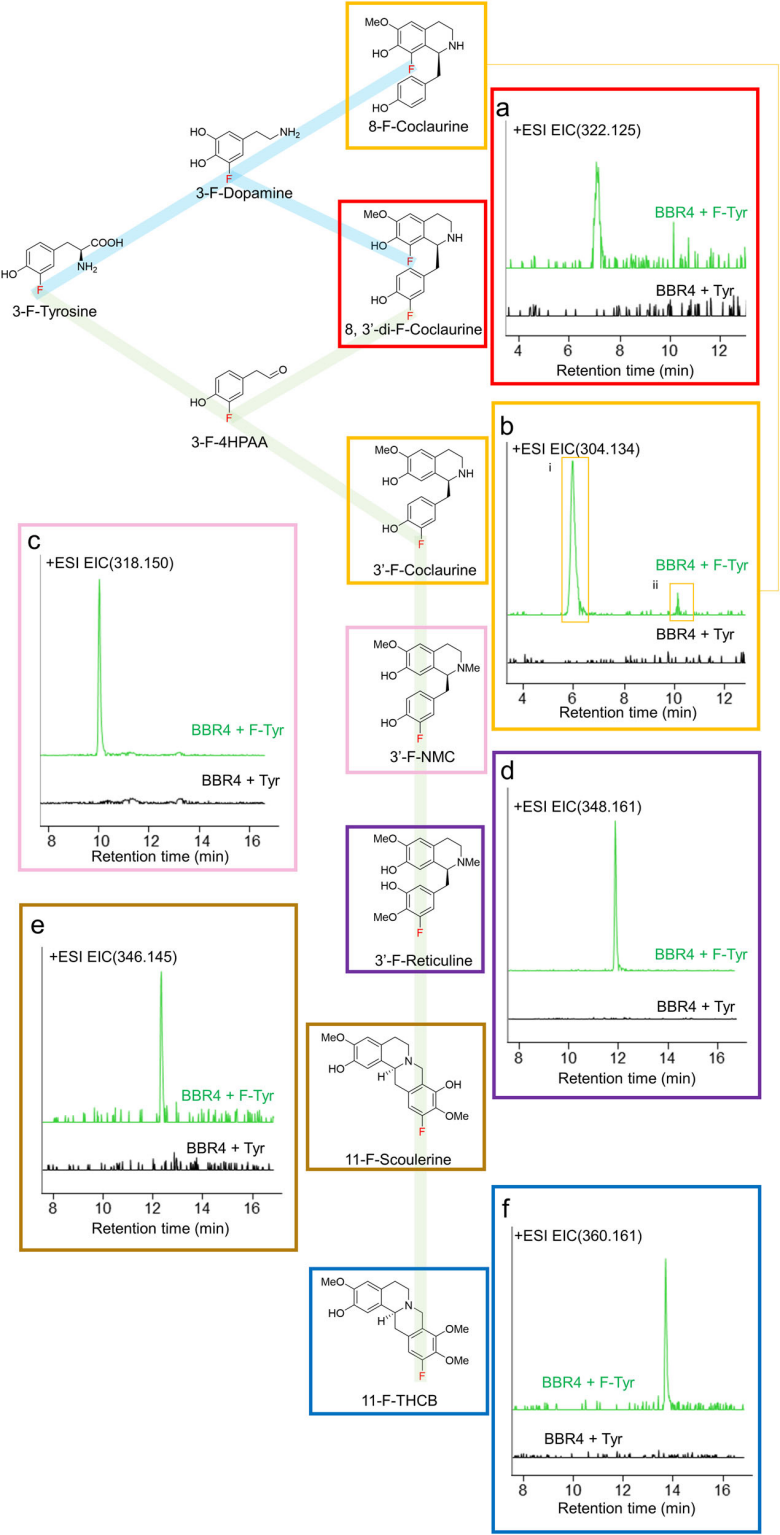
Production of F-substituted BIAs in BBR4R from 3-F-tyrosine feeding. Schema of proposed semi-biosynthetic pathway of F-BIAs from 3-F-tyrosine. Dopamine route (blue line) and 4HPAA route (green line) are shown. The high-resolution EIC of (**a**) 8, 3’-di-F-Coclaurine, (**b**) i) 8-F-Coclaurine, ii) 3’-F-Coclaurine, (**c**) 3’-F-NMC, (**d**) 3’-F-Reticuline, (**e**) 11-F-Scoulerine, (**F**) 11-F-THCB. The EIC was extracted using the calculated m/z of target compounds (M + H)^+^, with a mass accuracy below 100 ppm. Traces are representative of three biological replicates.

Chemical modification such as halogenation can change the bioavailability of natural products and improve pharmaceutical activities^27^. Based on our berberine-production platform in yeast, we further explored its feasibility to produce halogenated BIA derivatives with higher pharmaceutical potentials. Strain BBR4R was cultured in 3 mL SD-Tyr medium supplemented with 100 mg L^−1^ 3-F-tyrosine as the substrate in tubes. After three days at 30 °C in tubes, the culture medium was analyzed by LC-MS. Two coclaurine derivatives with single F-substitution, later deduced as 8-F-coclaurine and 3’-F-coclaurine, were observed based on two peaks showing m/z = 304.134 under 50ppm at the retention time of 6.45 min and 10.23 min (Fig. 4b). Tandem mass spectrometry (MS/MS) determines the two peaks showed different fragmentation spectrums. Comparison of the two fragmentation spectrums and the spectrums of original coclaurine reflected two different structures in F-coclaurine (i) the F-substitution is on the isoquinoline ring, or F-coclaurine (ii) the F-substitution is on the benzyl ring, respectively (Supplementary Fig. S5). The results indicate that 3-F-tyrosine were incorporated into the BIA skeleton via two precursors respectively, either via 3-F-dopamine to the isoquinoline ring leading to 8-F-coclaurine, or via 3-F-4HPAA to the benzyl ring leading to 3’-F-coclaurine (Fig. 4 and Supplementary Fig. S4). Only the 3’-F-coclaurine was incorporated into the downstream pathway towards 3’-F-N-methylcoclaurine (3’-F-NMC, m/z 318.150) and 3’-F-reticuline (m/z 348.161), identified respectively by Extracted ion chromatogram (EIC) at 50 ppm and MS/MS (Fig. 4c, d and Supplementary Fig. S6, S7). It indicates that CNMT, the enzyme after coclaurine, might prefer the F-BIAs produced from the 3-F-4HPAA route. The 3’-halogenation sites of N-methylcoclaurine (NMC) and reticuline are different from that reported in the previous study at the 8- site^22^. The BBR4R strain constructed in this work favors the 3-F-4HPAA route to the 3-F-dopamine route, which is likely due to the deletion of four 4HPAA degrading genes and the substrate preference of the *B. vulgaris* TyrH enzyme. We found the peaks with m/z of F-scoulerine (m/z 346.145, 50ppm) and F-THCB (m/z 360.161, 50ppm), presumably 11-F-scoulerine and 11-F-THCB (Fig. 4e, f and Supplementary Fig. S8, S9), while the previous study only observed halogenated reticuline^22^ although the substrate promiscuity of downstream enzyme BBE was reported in vitro^51,52^. This finding is the first evidence to prove that the halogenated BIAs can be incorporated into the protoberberine pathway downstream of reticuline in vivo and that the promiscuity of BBE is able to accept 3’-F-reticuline to synthesize downstream BIAs. The retention time of all F-BIAs were approximately 1-min longer than their corresponding original BIAs, indicating increased hydrophobicity, which supported the identification of these F-BIAs, as fluorocarbon was reported more hydrophobic than hydrocarbon^53,54^. The hydrophobicity of F-BIAs can contribute to improve drug transport in human body, such as enhanced blood–brain barrier permeability^55^.

Surprisingly, we also observed a peak that matches the m/z of 8, 3’-di-F-coclaurine (322.125, 50 ppm) (Fig. 4a) and identified its structure by MS/MS, which indicated fluorine in both the isoquinoline and the benzyl rings (Supplementary Fig. S5). It showed that the enzyme NCS, which condensates the two precursors dopamine and 4HPAA, has a promiscuity to condensate 3-F-dopamine and 3-F-4HPAA simultaneously, despite that the peak area of di-F-coclaurine is only 2% of the single substituted BIAs.

We also tested the incorporation of 3-Cl-tyrosine and 3-I-tyrosine in this pathway and achieved the production of 3’-Cl-NMC, 3’-Cl-reticuline and 3’-I-NMC (Supplementary Fig. S10). The less efficient incorporation of Cl and I in the pathway may be due to the larger steric hindrance.

Finally, we confirmed the scalability of F-BIA production in 0.75 L bioreactor fermentation. Quantification of the end products of the three routes showed that batch fermentation in a bioreactor increased F-BIA synthesis significantly. 8, 3’-di-F-coclaurine and 8-F-coclaurine (after four enzymatic transformations) were increased by 1.6-fold and 1.9-fold, respectively, estimated by the relative EIC peak areas. 11-F-THCB (after nine enzymatic transformations) were increased by 17-fold, indicating the bioreactor fermentation optimizes this route most (Supplementary Table S2). The production of these halogenated BIAs with different halogen groups at different sites highlights the capability of the reconstructed pathway in yeast to serve as a platform for manufacturing novel BIA derivatives by the substrate-feeding approach, offering an alternative way for drug discovery and development.

## Discussion

In this study, we demonstrated the complete biosynthesis of berberine in yeast. The engineered yeast strain contains 12 heterologous enzymes from plant or bacteria, along with the overexpression of three yeast endogenous enzymes to enhance the supply of tyrosine and the deletion of four yeast endogenous enzymes to lower the competition for the upstream precursor 4HPAA. With the engineering of 19 genes and chemical conversion by heating, the berberine production reached 1.08 mg L^−1^ in bioreactor fermentation, which highlights the feasibility of yeast fermentation to serve as an industrial supply chain for plant natural products with further improvement including enzyme engineering, strain engineering and bioprocess optimization. Notably, several novel halogenated BIA compounds, including 3’-F-scoulerine, 11-F-THCB and 8, 3’-di-F-coclaurine, were produced in yeast from 3-F-tyrosine fed during the fermentation, which highlights the value of yeast fermentation to provide tailor-made BIA derivatives for drug development.

Oxidative aromatization is the final step to synthesize berberine, sanguinarine and papaverine, all with pharmaceutical potentials. In vitro biochemical validations have proven that all these reactions are catalyzed by a family of FAD-dependent oxidoreductases in plants, including STOX in berberine synthesis^46^ and dihydrobenzophenanthridine oxidase (DBOX) in sanguinarine^50^ and papaverine synthesis. However, the in vivo activities of corresponding oxidoreductases remain difficult to validate in heterologous hosts such as yeast^32,56^. In particular, the in vivo activity of CjSTOX from *C. japonica* in berberine synthesis was not observed in yeast, *E. coli*, or in California poppy (side reactions converting cheilanthifoline to dehydrocheilanthifoline and stylopine to coptisine were observed)^49^. In our work, we confirmed CjSTOX’s activity in converting canadine to berberine by transient expression in the model plant *N. benthamiana*. CjSTOX showed marginal or no activity in yeast system despite of various engineering efforts. Engineered CjSTOX variants bound to yeast ER membrane, vacuole membrane, or inside vacuole did not improve berberine production, although STOX was reported to localized on ER and vacuole in plant cells^57,58^. Lack of cofactor used to be a common bottleneck hindering the functional expression of heterologous plant enzymes in yeast, yet we postulate that FAD supply is abundant in the engineered yeast, as another FAD-dependent oxidoreductase, BBE, remains active in yeast. Future studies investigating other possibilities might address this challenge. For example, the involvement of auxiliary proteins or the intercellular transportation^59–61^ of BIAs in plant might be essential for berberine synthesis.

Chemical oxidation provides an alternative solution to address the inactivity of STOX in yeast. As the berberine production during fermentation is likely due to the spontaneous conversion, we hypothesized and proved that heating and higher oxygen transfer in bioreactor can increase the conversion by 29.8-fold from 35.1 μg L^−1^ to 1.08 mg L^−1^. A recent publication of papaverine biosynthesis reported the similar bottleneck in which the FAD-dependent oxidoreductase DBOX is inactive in yeast and used a similar strategy to convert tetrahydropapaverine to papaverine using peroxide and heating condition^56^. Semi-biosynthesis has proven a feasible approach in the production of valuable and challenging plant natural products such as artemisinin and vinblastine^16,19^. Both our chemical conversion using heating and the reported conversion combining peroxide and heating proved that a mild chemical conversion can compensate for the lack of functional enzyme as a versatile and convenient method for future industrial applications.

Structure modification including halogenation on natural products is a common approach to improve their bioavailability and bioactivity^27,62^. Modified BIAs have been a group of attractive targets in the recent years because of their wide pharmaceutical properties. Enhanced bioactivity of halogenated BIAs, such as chloro-berberine on glucose uptake^63^ and fluoro-aporphine on neurotransmitter receptors^64,65^, has been reported, showing the huge potential of halogenated BIAs in drug design. In the previous study of synthesizing noscapine in yeast, 8-F-reticuline and 8-Cl-reticuline were produced from 3-F/Cl-tyrosine via seven in vivo enzymatic transformations^22^. When we are preparing this manuscript for submission, another group reported an in vitro enzymatic approach to synthesize a diverse group of halogenated norcoclaurine and coclaurine derivatives by using different substrates in four to five cascaded reactions^31^. The utilization of a set of different plant enzymes and pathway optimization strategy enable us to extend the incorporation of 3-F-tyrosine beyond reticuline in vivo towards halogenated 11-F-THCB (nine enzymatic transformations in total). This achievement has showcased the capacity of in vivo incorporation of halogenated intermediates in the longer pathway. Further enzyme engineering efforts focusing on the substrate binding site of CAS will help address the last missing enzymatic transformation to produce downstream F-canadine and F-berberine.

Most halogenated BIAs produced in this study were characterized by LC-MS/MS as 3’-F-BIAs, indicating F-tyrosine was incorporated via the 4HPAA route rather than the dopamine route in the previous noscapine-producing strain^22^. The preference of 4HPAA route is likely due to the deletion of four yeast endogenous genes related to 4HPAA degradation and the use of the plant-derived TyrH instead of mammalian TyrH. The difference showed a possibility to control the halogenation site by tuning the metabolic flux balance between 4HPAA and dopamine route. More flux in the 4HPAA routes would lead to higher fraction of 3’-F-BIA, while 8-F-BIA would be the majority for the opposite condition. Moreover, the balance may impact the production of di-F-BIAs that requires F-tyrosine to be incorporated via both routes simultaneously. In our study, both 3’-F and 8-F halogenation was observed for the upstream compound coclaurine, together with 8, 3’-di-F-coclaurine, which is the first di-fluoro BIA compound synthesized in yeast. No further 8-F and di-F BIAs were observed, which is maybe due to the lack of F-BIA flux via the dopamine routes. An optimized strain with balanced upstream flux of dopamine and 4HPAA routes will lead to the production of more di-F-BIAs, which can significantly contribute to the new drug discovery and development.

## Methods

### Chemicals, genes, kits, and oligonucleotides

Yeast nitrogen base (YNB) and amino acid mixtures were purchased from Sunrise Science Products. Ammonium sulfate, dithiothreitol (DTT), 3-F-tyrosine, 3-Cl-tyrosine, 3-I-tyrosine, berberine standard, and canadine standard were purchased from Sigma-Aldrich. All other chemicals were purchased from VWR International or Fisher Scientific.

Coding sequences of the heterologous genes (listed in Supplementary Data 2) were codon-optimized for *S. cerevisiae* expression and synthesized by TWIST Bioscience. Oligonucleotide primers (listed in Supplementary Data 3) were synthesized by Life Technologies.

Q5 High-Fidelity 2X Master Mix, Gibson Assembly Master Mix, DNA ladder, and gel loading dye were purchased from New England Biolabs. Gateway LR Clonase II Enzyme mix was purchased from Life Technologies. Kits for plasmid miniprep, gel DNA recovery, *E. coli* transformation, yeast genome extraction, and frozen-EZ yeast transformation were purchased from Zymo Research.

### Plasmid construction

Plasmids used in this study is listed in Supplementary Table S1. Heterologous genes were assembled on the holding plasmids by Gibson Assembly^66^ using Gibson Assembly Master Mix. Synthesized genes were amplified by PCR using the Q5 DNA polymerase. Holding plasmid backbones (pE series) with designed promoter and terminator pairs were also amplified by PCR. PCR products were verified by agarose gel electrophoresis and purified with a gel DNA recovery kit following the manufacturer’s instructions. Plasmids for yeast expression (pAG series^47^) and plant transient expression (pCambia2300 series) were constructed using Gateway LR Clonase II Enzyme mix.

*E. coli* competent cells were prepared using an *E. coli* transformation kit following the manufacturer’s instructions. *E. coli* strains harboring plasmids were cultured in LB media at 37 °C with 50 μg mL^−1^ of kanamycin or 100 μg mL^−1^ of carbenicillin as appropriate. Plasmids were extracted using a plasmid miniprep kit according to the manufacturer’s instructions, followed by concentration measurement through NanoDrop One (ThermoFisher Scientific) and Sanger Sequencing (Cornell Institute of Biotechnology or GENEWIZ).

### Yeast strain construction

Yeast genome integrations were performed by electroporation as previously reported^40^. Pathway genes, selection markers and 500 bp genomic homologies were amplified by PCR from holding plasmids or yeast genome with 30–40 bp overlaps between adjacent fragments. Amplified DNA fragments were verified by agarose gel electrophoresis, purified from agarose gel slices, and then transformed at equimolar ratios into yeast by electroporation, at 540 V, 25 μF and infinite resistance with a Gene Pulser Xcell Total System electroporator (Bio-Rad). Cells were then recovered immediately in 1 mL YPD at 30 °C for 2 h and spread on selection plates for 2–4 days of growth. If necessary, the integration selection marker was then rescued by expression of Cre recombinase on plasmid^45^, followed by replica printing selection on SD plates and 48-hour culture in YPD to rescue the Cre plasmid.

For plasmid expression, chemically competent yeast cells were prepared using frozen-EZ yeast transformation II kits. 200 ng of plasmids were used for each transformation following the manufacturer’s instructions. Additionally, 2-hour outgrowth in 1 mL YPD at 30 °C was applied if the selection marker is HygR. Cells after transformation were spread on selection plates for 2–4 days of growth.

### Culture and tube fermentation conditions

For berberine production, yeast colonies were first cultured in 14 mL falcon tubes with 1 mL YPD (1% yeast extract, 2% peptone, 2% dextrose) at 30 °C overnight. 150 μL cultures were then diluted in 3 mL fresh YPD and cultured for 3 days in triplicates. If necessary, 200 mg L^−1^ hygromycin was supplemented to YPD for selection. For halogenated BIA production, culture medium is substituted with SD-tyr (0.17% yeast nitrogen base, 0.5% ammonium sulfate, and 1× -tyr amino acid drop-out mixture) supplemented with 2% dextrose and 100 mg L^−1^ halogenated tyrosine. After fermentation, 300 μL of each culture was harvested and centrifuged at 15,000 rpm for 10 min, and then 200 μL supernatant was transferred to Agilent 96-well plate for LC-MS analysis.

To further increase berberine production via chemical oxidation, culture supernatant was transferred to PCR tubes, 100 μL per tube, and heated at 98 °C in a thermocycler.

### Plant screening of STOX

Sequences of STOX candidates were assembled on the pCambia2300 plasmid by Gateway LR reaction. Confirmed constructs were then transformed into *A. tumefaciens* GV3101 via the freeze-thaw method^48^ and spread on LB agar with appropriate antibiotics at 30 °C for two days. Single colonies were cultivated in four mL LB with 100 μg mL^−1^ carbenicillin, 25 μg mL^−1^ rifamycin and 30 μg mL^−1^ gentamycin for 24 h and then diluted in 100 mL for another 24 h. Cells were pelleted by centrifuge at 4000 rpm for 15 min and resuspended in 100 mL induction buffer (10 mM MES, pH 5.6, 10 mM MgCl2, 150 μM acetosyringone), incubated at 30 °C for 3 h. Induced cells were pelleted again and diluted in induction buffer to an OD600 of 0.6. Suspensions were then infiltrated into the abaxial side of *N. benthamiana* leaves of 4-week-old plants using needleless 1-ml syringes, approximately 0.5–1 mL per leaf. Three individual leaves from different plants were used as replicates for each construct. After four days for gene expression, 1 mg L^−1^ canadine dissolved in water was infiltrated into the abaxial side as substrate, approximately 0.5–1 mL per leaf. Leaves were excised 1 day later and stored in −80 fridge for downstream processing.

Frozen leaves were lyophilized and weighted for dry mass. 80% (v/v) methanol solution with 0.1% (v/v) formic acid was used to extract the metabolites, with 10 μL per mg dry weight. Then they were homogenized by a Benchmark BeadBug 6 homogenizer with 3 mm beads. Extracted samples were filtered through a Pall AcroPrep 96-well filter to an Agilent 96-well plate for LC-MS analysis.

### Bioreactor fermentation conditions

For berberine production, fresh single colonies on plate were first cultured in tubes with 2 mL YPD at 30 °C overnight as the first seed cultures. Then 1.25 mL of each culture was used to inoculate 2*25 mL fresh YPD in 2*250 mL shake flasks and cultured at 30 °C overnight, as the second seed cultures. Subsequently, the second seed cultures were inoculated to 0.75 L YPD in a 2.5 L Eppendorf BioFlo 310 vessel. Fermentations were performed at 30 °C, 600 rpm. The pH was automatically adjusted at 7.0 using 1 M NaOH solution. At every sampling time point, 1 mL culture was harvested, measured OD600 by Nanodrop, and then centrifuged for LC-MS analysis.

### Metabolites LC-MS and LC-MS/MS analysis

Metabolites were assayed by high-resolution liquid chromatography coupled with quadrupole time-of-flight mass spectroscopy (Agilent 1260 Infinity II/Agilent G6545B) in MS mode using positive ionization. 1 μL of each sample was injected and separated in the ZORBAX RRHD Eclipse Plus C18 column (2.1 × 50 mm, 1.8 μm) (Agilent) with water with 0.1% formic acid (A) and acetonitrile with 0.1% formic acid (B) as the mobile phases. The gradient program of the binary pump was set as 0–1 min, 95% A; 1–11 min, 95–5% A; 11–13 min, 5% A; 13–14 min, 5–95% A; and 14–16 min, 95% A at a flow rate of 0.4 mL min^−1^. For detecting halogenated compounds, a longer gradient program was used: 0–4 min, 95% A; 4–44 min, 95–5% A; 44–52 min, 5% A; 52–56 min, 5–95% A; and 56–60 min, 95% A at a flow rate of 0.4 mL min^−1^. The m/z values of the [M + H]^+^ adduct were calculated by ChemDraw software and then used to extract the ion chromatogram (with a mass error below 50 ppm) for compound identification and quantification. For identification of halogenated BIAs, tandem mass spectrometry (MS/MS) mode with three different levels of collision energy (10, 20 and 40 eV) was used and the inject volume was adjusted to 5 μL.

### Statistics

Three independent biological replicates were used in the experiments of Fig. 2c–f and Supplementary Fig. S3a. Two-tailed *t*-test was applied to calculate the *p*-value in Fig. 2c, d. Error bars represent standard deviation. For the experiment of Fig. 3, error bars represent either standard deviation of three independent fermentations for points before 72 h or the range of two independent fermentations for points after 72 h. EIC traces and MS spectrums shown in Figs. 2a, b and 4, and Supplementary Figs. S1, S4, and S5 are represented for three to five independent replicates.

### Softwares

Figures were generated through Prism9 (Graphpad) and PowerPoint 2019 (Microsoft) whatever necessary. MassHunter Workstation (Agilent) was used to collect and analyze LC-MS and LC-MS/MS data. Chemical structures were generated through ChemDraw 20.1 (PerkinElmer).

### Reporting summary

Further information on research design is available in the Nature Portfolio Reporting Summary linked to this article.

## Supplementary information

Supplementary information

Description of Additional Supplementary Files

Supplementary Data 1

Supplementary Data 2

Supplementary Data 3

Reporting Summary

## Data Availability

The authors declare that source data processed for figure generation in this study are available within the paper and its Supplementary Information files. The source data underlying Figs. 2c–f, 3, and Supplementary Fig. S3a are provided as a Supplementary Data 1. Gene sequences used in this study are deposited in Supplementary Data 2. Primer sequences used in this study are deposited in Supplementary Data 3. The datasets generated and analyzed during the current study are available from the corresponding authors upon request.
