# Supplementary material for: De novo biosynthesis of berberine and halogenated benzylisoquinoline alkaloids in Saccharomyces cerevisiae
Source: Commun Chem. 2023 Feb 9;6:27. doi: 10.1038/s42004-023-00821-9 (PMC9911778; doi:10.1038/s42004-023-00821-9)
Supplement: Supplementary file 1 — Supplementary information [file 42004_2023_821_MOESM1_ESM.pdf]

Supplementary Info for

De novo biosynthesis of berberine and halogenated benzylisoquinoline alkaloids in *Saccharomyces cerevisiae*

Authors:

Jianing Han[a], Sijin Li[a,*]

Affiliations:

[a] Robert F. Smith School of Chemical and Biomolecular Engineering, Cornell University, Ithaca, NY, 14853, USA

Co-corresponding authors*:

E-mail address: sijin.li@cornell.edu (S. Li).

Supplementary Method

Fluorescence microscopy

Wild-type CjSTOX or engineered CjSTOX with signal peptide, which relocalizes the enzyme to ER or vacuole (listed in Supplementary table S2), were fused with mCherry fluorescence protein and constructed on pAG42H vector. Individual colonies of yeast strains with the constructs were cultured in 3 mL YPD with 200 mg/L hygromycin for three days. Approximately 5 µL cell culture was spotted onto a glass microscope slide and covered with a glass coverslip. Glass slides were imaged using a Zeiss LSM710 confocal microscope with a × 64 oil immersion objective. Subsequent image analysis was performed in ZEN (Zeiss).

Supplementary Table S1. Strains and plasmids used in this study

	Description	Reference
**Holding plasmid backbone and marker templates**		
pSL1	P_{HXT7}/T_{PGK1}	Smolke lab
pSL2	P_{TPI1}/T_{STE2}	Smolke lab
pSL3	P_{GPD1}/T_{ADH1}	Smolke lab
pSL4	P_{TEF1}/T_{CYC1}	Smolke lab
pSL5	P_{PGK1}/T_{PHO5}	Smolke lab
pSL9	P_{PYK1}/T_{MFA1}	Smolke lab
**Gene cassette holding plasmids**		
pSL29	P_{TEF1}-AtATR1-T_{CYC1}	Smolke lab
pSL315	P_{HXT7}-yBvTyrH-T_{PGK1}	This study
pSL316	P_{TPI1}-yPpDoDC-T_{STE2}	This study
pSL317	P_{GPD1}-yCjNCS(N35)-T_{ADH1}	This study
pSL318	P_{PGK1}-yPs6OMT-T_{PHO5}	This study
pSL319	P_{GPD1}-yPsCNMT-T_{ADH1}	This study
pSL320	P_{HXT7}-yEcNMCH-T_{PGK1}	This study
pSL321	P_{TEF1}-yPs4'OMT-T_{CYC1}	This study
pSL322	P_{TEF1}-yPsBBE-T_{CYC1}	This study
pSL323	P_{PYK1}-yPsS9OMT-T_{MFA1}	This study
pSL324	P_{HXT7}-yCjCAS-T_{PGK1}	This study
pSL325	P_{GPD1}-yBwSTOX-T_{ADH1}	This study
pSL326	P_{GPD1}-yCjSTOX-T_{ADH1}	This study

Supplementary Table S1. (Continued)

	Description	Reference
**pCambia2300 plasmids for transient expression in plant**		
pSL330	pCambia2300-eGFP	This study
pSL358	pCambia2300-BwSTOX	This study
pSL359	pCambia2300-CjSTOX	This study
pSL360	pCambia2300-PsDBOX	This study
**pAG plasmids for overexpression in yeast**		
pSL196	2µ vector, Leu2 marker, gateway cloning site	[1]
pSL357	2µ vector, HygR marker, gateway cloning site	This study
pSL361	2µ vector, HygR marker, P_{GPD1}-yBwSTOX-T_{ADH1}	This study
pSL362	2µ vector, HygR marker, P_{GPD1}-yCjSTOX-T_{ADH1}	This study
pSL363	2µ vector, HygR marker, P_{GPD1}-yCjSTOX-mCherry-T_{ADH1}	This study
pSL364	2µ vector, HygR marker, P_{GPD1}-yCjSTOXΔN35-T_{ADH1}	This study
pSL365	2µ vector, HygR marker, P_{GPD1}-yCjSTOX (MNS1SP)-T_{ADH1}	This study
pSL366	2µ vector, HygR marker, P_{GPD1}-yCjSTOX (PEP4SP)-T_{ADH1}	This study
pSL222	Cre overexpression plasmid for marker rescue. Trp1 marker.	[2]
**Bacteria strains**		
E.coli Top10	For plasmid construction	ATCC
Agrobacteria GV3101	For agrobacteria infiltration	ATCC
**Yeast strains**		
CEN.PK2-1D	MATα; ura3-52; trp1-289; leu2-3;112 his3 Δ1; MAL2-8C; SUC2;	EUROSCARF
ySL14	CEN.PK-1D, ΔYBL059W::P_{ARO4}-ScARO4^{Q116K}-T_{ARO4}/P_{ARO7}-ScARO7^{T226I}-T_{ARO7}/P_{GPM1}-KpHYGR-T_{PYK1}/P_{TEF2}-ScTKL-T_{FBA1}	[3]
BBR1 (ySL68)	ySL14, ΔYGL157W::P_{HXT7}-yBvTyrH-T_{PGK1}/P_{TPI1}-yPpDoDC-T_{STE2}/P_{AgTEF}-SpHIS5-T_{AgTEF}/P_{GPD1}-yCjNCS(N35)-T_{ADH1}/P_{TEF1}-AtATR1-T_{CYC1}	This study
BBR2 (ySL69)	BBR1, ΔYMR318C::P_{PGK1}-yPs6OMT-T_{PHO5}/P_{GPD1}-yPsCNMT-T_{ADH1}/P_{KlLEU2}-KlLEU2-T_{KlLEU2}/P_{HXT7}-yEcNMCH-T_{PGK1}/P_{TEF1}-yPs4'OMT-T_{CYC1}	This study
BBR3	BBR2, ΔYDR368W::P_{TEF1}-yPsBBE-T_{CYC1}/P_{PYK1}-yPsS9OMT-T_{MFA1}/P_{KlURA3}-KlURA3-T_{KlURA3}/P_{HXT7}-yCjCAS-T_{PGK1}/P_{GPD1}-yBwSTOX-T_{ADH1}	This study
BBR3R (ySL70)	BBR3, his5, leu2, ura3 marker rescued.	This study
BBR4	BBR3R, ΔYDR541C::P_{GPD1}-yCjNCS(N35)-T_{ADH1}/P_{TEF1}-yPs4'OMT-T_{CYC1}/P_{KlLEU2}-KlLEU2-T_{KlLEU2}/P_{HXT7}-yCjCAS-T_{PGK1}	This study
BBR4R (ySL71)	BBR4, hygR, leu2 marker rescued.	This study

Supplementary Table S2. Comparison of F-BIAs titers in bioreactor or tube

	m/z	Peak area of tube samples	Peak area of bioreactor samples	fold increase
8-F-coclaurine	304.134	558913.22	1642327.07	1.9
8, 3'-di-F-coclaurine	322.125	84471.03	221498.36	1.6
3'-F-THCB	360.161	205028.62	3687348.43	17.0

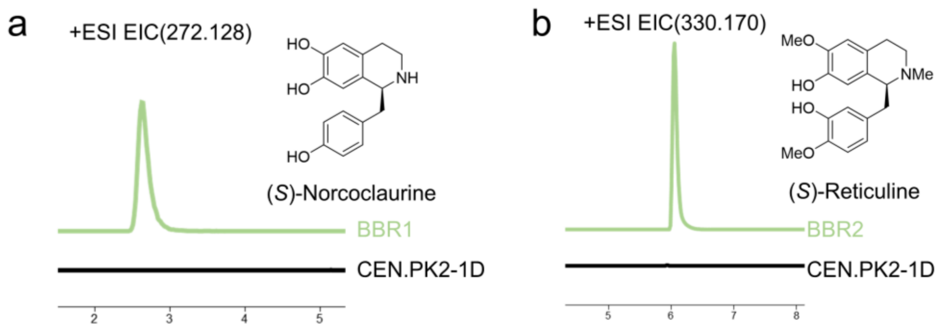

Supplementary Figure S1. Production of **a**) norcoclaurine in BBR1, and **b**) reticuline in BBR2. EIC trace are representative of three biological replicates.

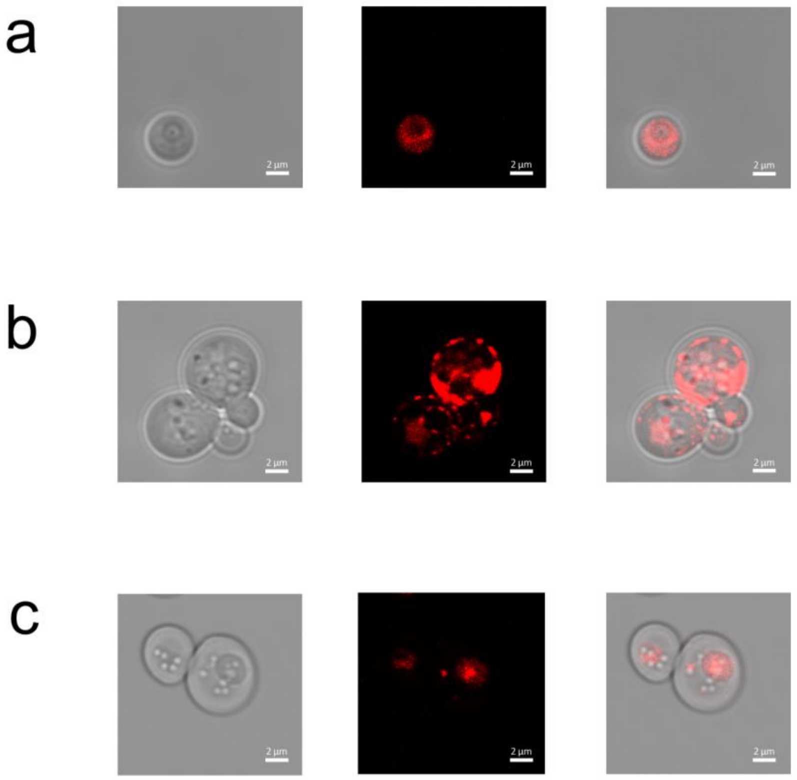

Supplementary Figure S2. Fluorescence microscopy image of yeast cells expressing **a**) wild-type CjSTOX, **b**) ER-targeted Mns1sp-CjSTOX, or **c**) vacuole-targeted Pep4sp-CjSTOX, fused with mCherry fluorescence protein respectively. Left, bright field; Middle, mCherry fluorescence; Right, overlapped.

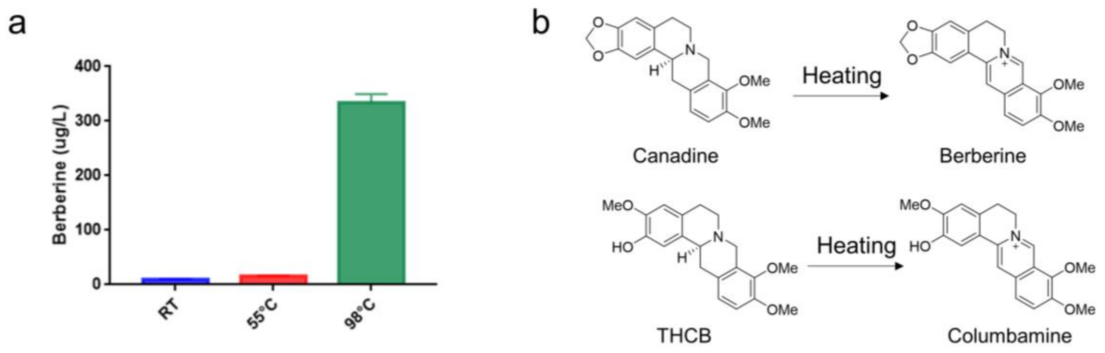

Supplementary Figure S3. Heating treatment helped berberine conversion. **a**) Berberine conversion from pure canadine standard under different heating conditions. Error bars represent standard deviation of three independent replicates. **b**) Proposed two conversions in the heating of BBR4R culture.

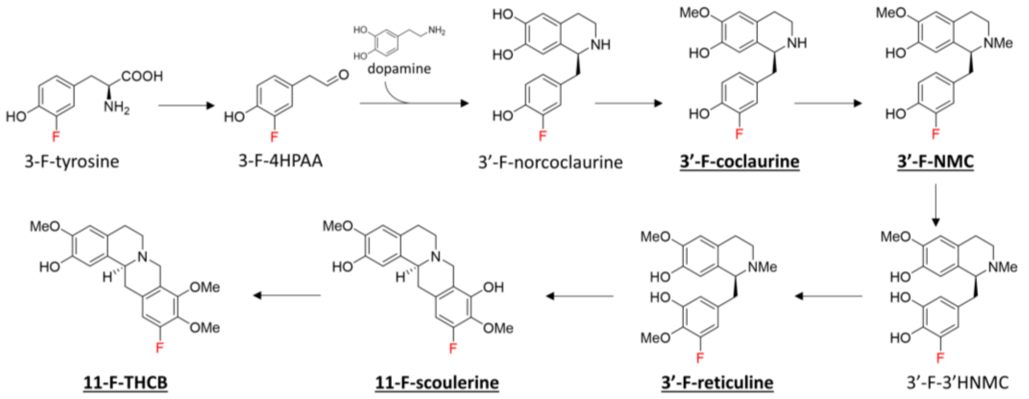

Supplementary Figure S4. Complete schema of 3-F-tyrosine incorporation to BIA structures via the 4-HPAA route. Fluorine atoms are shown in red. Compounds with underlines have been identified in both MS and MS/MS.

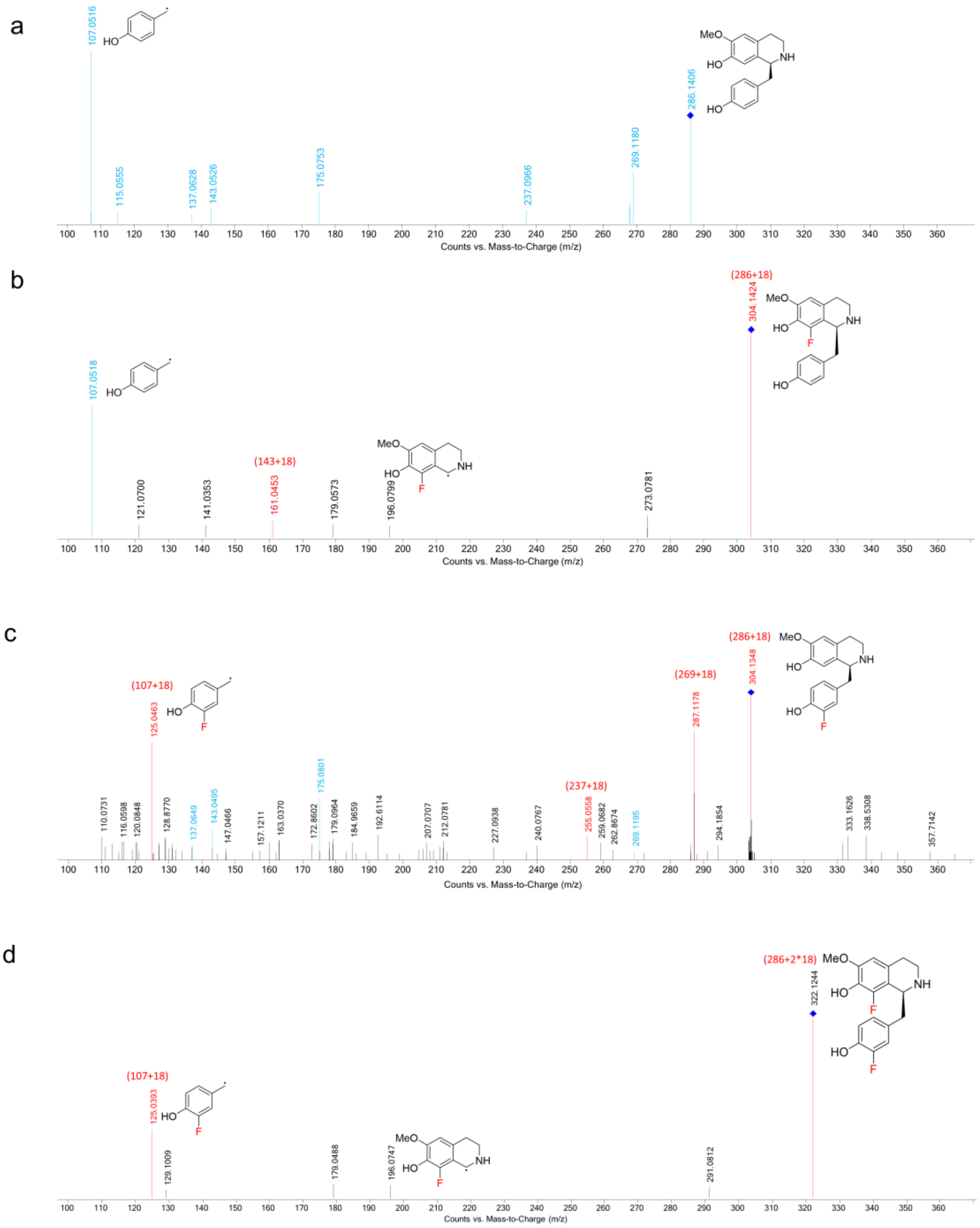

Supplementary Figure S5. Comparative analysis of MS/MS spectrum of **a**) coclaurine, **b**) 8-F-coclaurine, **c**) 3’-F-coclaurine, **d**) 8, 3’-di-F-coclaurine. Original coclaurine spectrum is all labeled in blue. Signals that keep the same m/z as original signals, indicating the same fragments, are labeled in blue. Signals that showed a m/z with an additional 18 compared with original signals, indicating fragments with an additional fluorine atom are labeled in red.

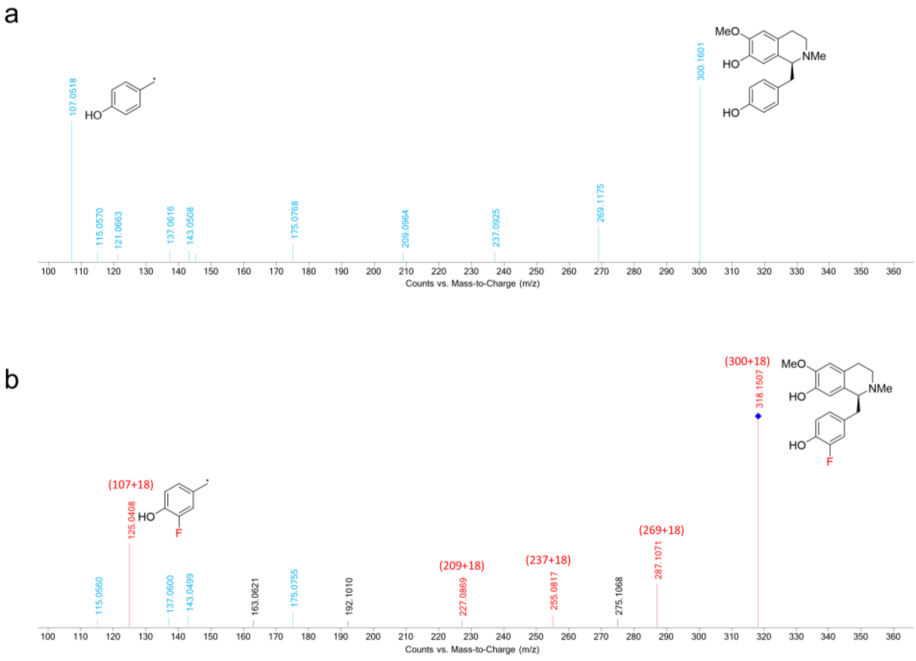

Supplementary Figure S6. Comparative analysis of MS/MS spectrum of **a**) NMC, **b**) 3’-F-NMC. Original NMC spectrum is all labeled in blue. Signals that keep the same m/z as original signals, indicating the same fragments, are labeled in blue. Signals that showed a m/z with an additional 18 compared with original signals, indicating fragments with an additional fluorine atom are labeled in red.

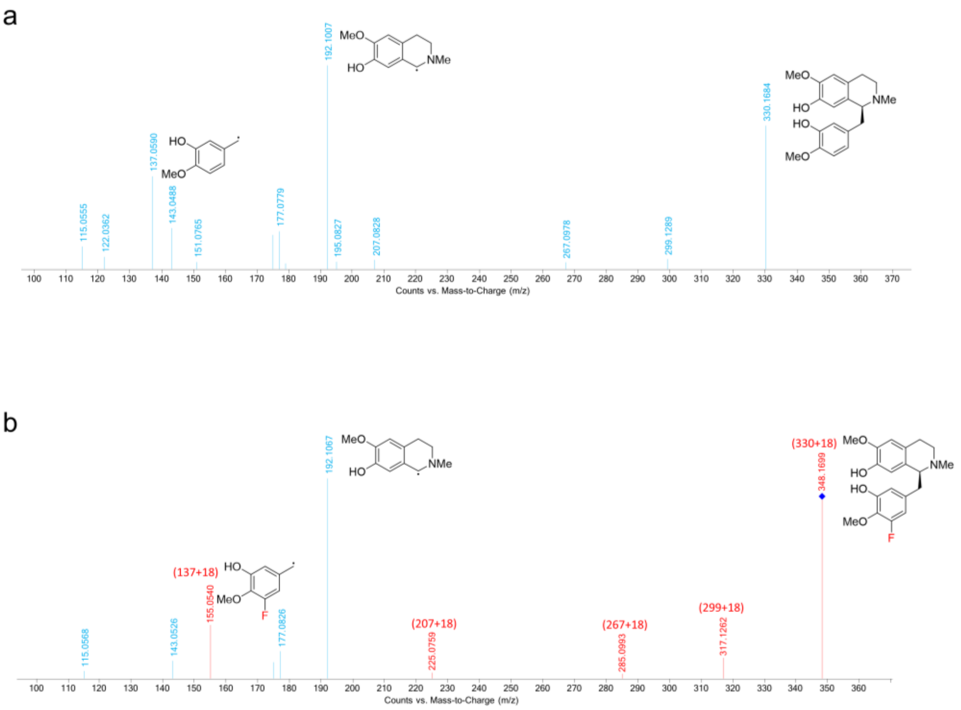

Supplementary Figure S7. Comparative analysis of MS/MS spectrum of **a**) reticuline, **b**) 3’-F-reticuline. Original reticuline spectrum is all labeled in blue. Signals that keep the same m/z as original signals, indicating the same fragments, are labeled in blue. Signals that showed a m/z with an additional 18 compared with original signals, indicating fragments with an additional fluorine atom are labeled in red.

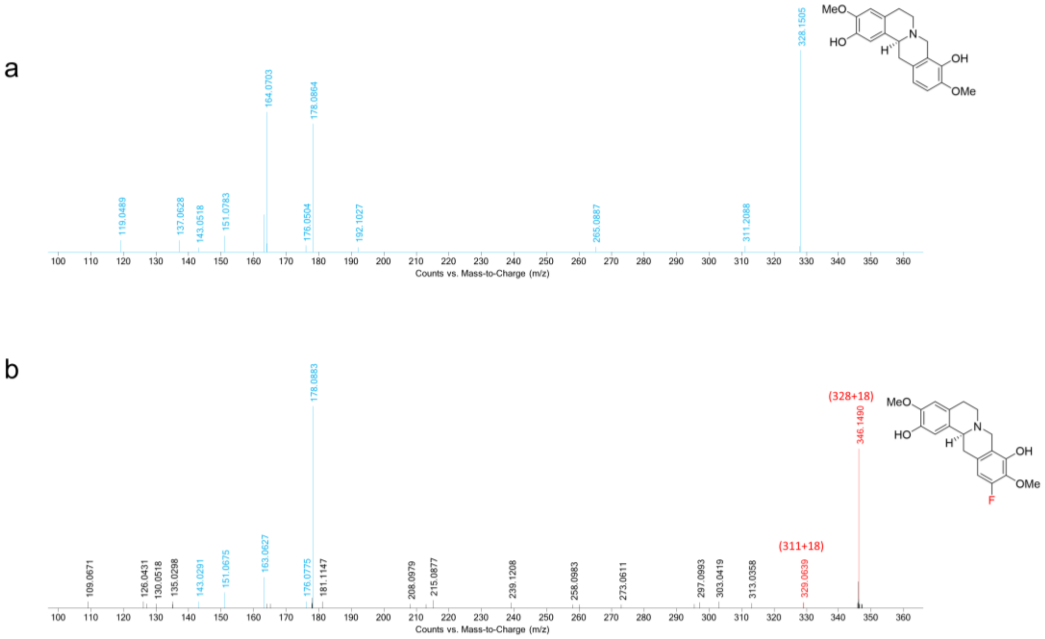

Supplementary Figure S8. Comparative analysis of MS/MS spectrum of **a**) scoulerine, **b**) 11-F-scoulerine. Original scoulerine spectrum is all labeled in blue. Signals that keep the same m/z as original signals, indicating the same fragments, are labeled in blue. Signals that showed a m/z with an additional 18 compared with original signals, indicating fragments with an additional fluorine atom are labeled in red.

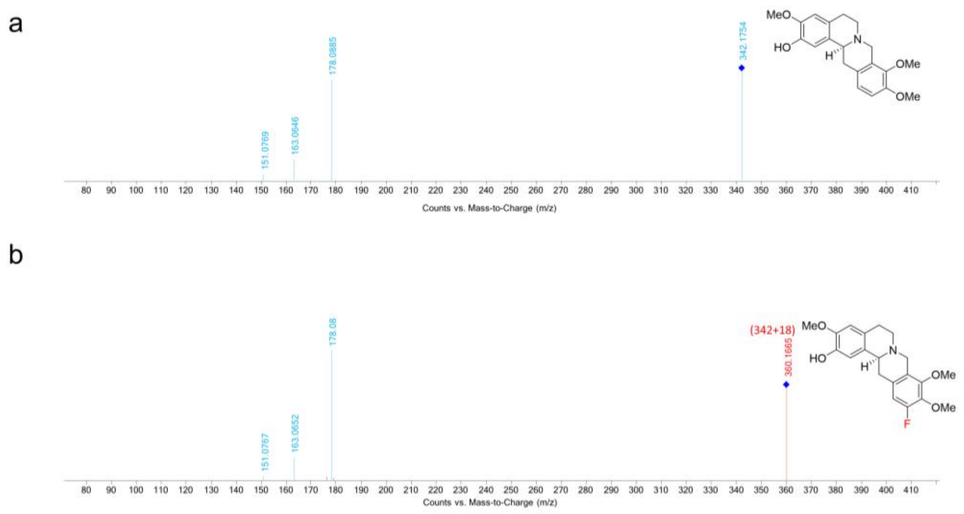

Supplementary Figure S9. Comparative analysis of MS/MS spectrum of **a**) THCB, **b**) 11-F-THCB. Original THCB spectrum is all labeled in blue. Signals that keep the same m/z as original signals, indicating the same fragments, are labeled in blue. Signals that showed a m/z with an additional 18 compared with original signals, indicating fragments with an additional fluorine atom are labeled in red.

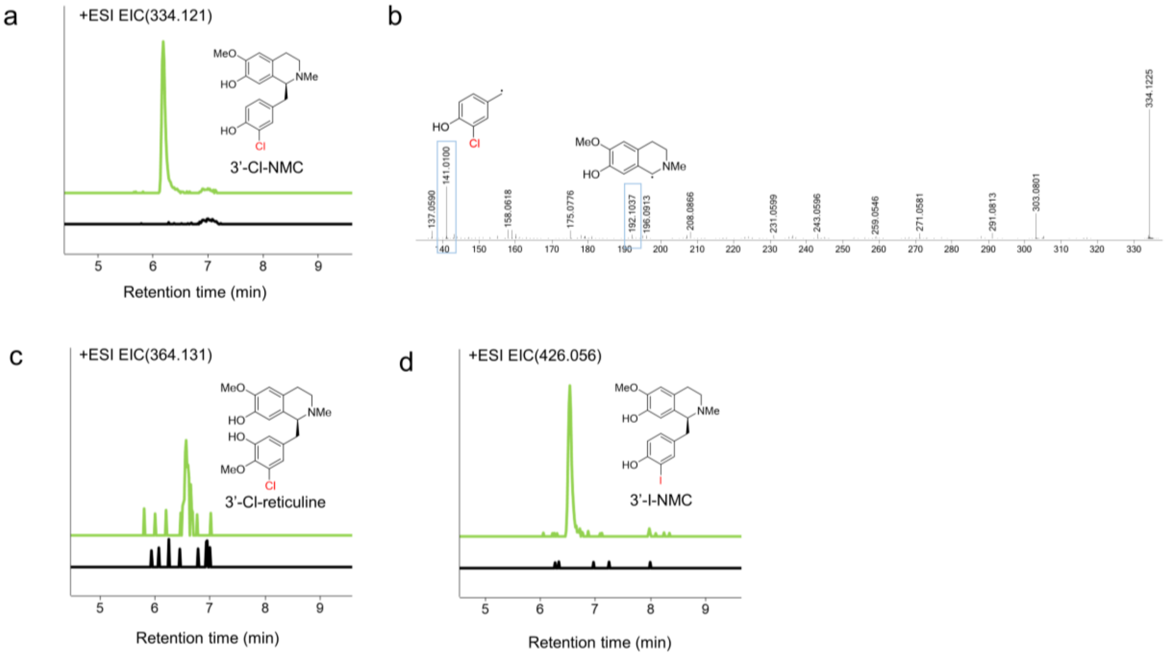

Supplementary Figure S10. Cl-substituted and I-substituted BIAs produced by BBR4 strain from 3-Cl-tyrosine or 3-I-tyrosine. **a**) EIC of 3’-Cl-NMC, **b**) MS/MS spectrum of 3’Cl-NMC, **c**) EIC of 3’-Cl-reticuline, **d**) EIC of 3’-I-NMC. Traces and spectrums are representative of three biological replicates.

Supplementary References

1. Gong, F. L., Han, J. & Li, S. MULTI-SCULPT: Multiplex Integration via Selective, CRISPR-Mediated, Ultralong Pathway Transformation in yeast for plant natural product synthesis. *ACS Synth Biol* **11**, 2484–2495 (2022).
2. Güldener, U., Heck, S., Fiedler, T., Beinhauer, J. & Hegemann, J. H. A new efficient gene disruption cassette for repeated use in budding yeast. *Nucleic Acids Res* **24**, 2519–2524 (1996).
3. Wu, Y., Chen, M. N. & Li, S. *De novo* biosynthesis of diverse plant-derived styrylpyrones in *Saccharomyces cerevisiae*. *Metab Eng Commun* **14**, (2022).

106
