# Supplementary material for: De novo biosynthesis of berberine and halogenated benzylisoquinoline alkaloids in Saccharomyces cerevisiae
Source: Commun Chem. 2023 Feb 9;6:27. doi: 10.1038/s42004-023-00821-9 (PMC9911778; doi:10.1038/s42004-023-00821-9)
Supplement: Supplementary file 2 — Description of Additional Supplementary Files [file 42004_2023_821_MOESM2_ESM.pdf]

Description of Additional Supplementary Files

File name: Supplementary Data 1

Description: The source data underlying Fig. 2c-f, Fig. 3, and Supplementary Fig. S3a

File name: Supplementary Data 2

Description: Gene sequences used in this study

File name: Supplementary Data 3

Description: Oligonucleotide primers
