# Supplementary material for: De novo biosynthesis of berberine and halogenated benzylisoquinoline alkaloids in Saccharomyces cerevisiae
Source: Commun Chem. 2023 Feb 9;6:27. doi: 10.1038/s42004-023-00821-9 (PMC9911778; doi:10.1038/s42004-023-00821-9)
Supplement: Supplementary file 6 — Reporting Summary [file 42004_2023_821_MOESM6_ESM.pdf]

nature portfolio

Corresponding author(s): Sijin Li

Last updated by author(s): Oct 3, 2022

Reporting Summary

Nature Portfolio wishes to improve the reproducibility of the work that we publish. This form provides structure for consistency and transparency in reporting. For further information on Nature Portfolio policies, see our Editorial Policies and the Editorial Policy Checklist.

Statistics

For all statistical analyses, confirm that the following items are present in the figure legend, table legend, main text, or Methods section.

n/a	Confirmed	
☐	☒	The exact sample size (n) for each experimental group/condition, given as a discrete number and unit of measurement
☐	☒	A statement on whether measurements were taken from distinct samples or whether the same sample was measured repeatedly
☐	☒	The statistical test(s) used AND whether they are one- or two-sided<br>*Only common tests should be described solely by name; describe more complex techniques in the Methods section.*
☒	☐	A description of all covariates tested
☒	☐	A description of any assumptions or corrections, such as tests of normality and adjustment for multiple comparisons
☐	☒	A full description of the statistical parameters including central tendency (e.g. means) or other basic estimates (e.g. regression coefficient) AND variation (e.g. standard deviation) or associated estimates of uncertainty (e.g. confidence intervals)
☐	☒	For null hypothesis testing, the test statistic (e.g. F, t, r) with confidence intervals, effect sizes, degrees of freedom and P value noted<br>*Give P values as exact values whenever suitable.*
☒	☐	For Bayesian analysis, information on the choice of priors and Markov chain Monte Carlo settings
☒	☐	For hierarchical and complex designs, identification of the appropriate level for tests and full reporting of outcomes
☒	☐	Estimates of effect sizes (e.g. Cohen's d, Pearson's r), indicating how they were calculated

Our web collection on statistics for biologists contains articles on many of the points above.

Software and code

Policy information about availability of computer code

Data collection	MassHunter Workstation (Agilent) was used to collect and analyze LC-MS and LC-MS/MS data.
Data analysis	Figures were generated through Prism9 (Graphpad) and PowerPoint 2019 (Microsoft) whatever necessary. MassHunter Workstation (Agilent) was used to collect and analyze LC-MS and LC-MS/MS data. Chemical structures were generated through ChemDraw 20.1 (PerkinElmer).

For manuscripts utilizing custom algorithms or software that are central to the research but not yet described in published literature, software must be made available to editors and reviewers. We strongly encourage code deposition in a community repository (e.g. GitHub). See the Nature Portfolio guidelines for submitting code & software for further information.

Data

Policy information about availability of data

All manuscripts must include a data availability statement. This statement should provide the following information, where applicable:

- Accession codes, unique identifiers, or web links for publicly available datasets
- A description of any restrictions on data availability
- For clinical datasets or third party data, please ensure that the statement adheres to our policy

The authors declare that source data processed for figure generation in this study are available within the paper and its Supplementary Information files. The source data underlying Fig. 2c-f, Fig. 3, and Supplementary Fig. S3a are provided as a Source Data file. Gene sequences used in this study are deposited in Supplementary Table S2. The datasets generated and analyzed during the current study are available from the corresponding authors upon request.

Human research participants

Policy information about studies involving human research participants and Sex and Gender in Research.

Reporting on sex and gender	N/A
Population characteristics	N/A
Recruitment	N/A
Ethics oversight	N/A

Note that full information on the approval of the study protocol must also be provided in the manuscript.

Field-specific reporting

Please select the one below that is the best fit for your research. If you are not sure, read the appropriate sections before making your selection.

☒ Life sciences ☐ Behavioural & social sciences ☐ Ecological, evolutionary & environmental sciences

For a reference copy of the document with all sections, see nature.com/documents/nr-reporting-summary-flat.pdf

Life sciences study design

All studies must disclose on these points even when the disclosure is negative.

Sample size	Sample size for all tube culture experiments were three, based on prior experience on similar experiments and reported research. Individual single colonies of each genotype were inoculated as the seed. The sample size of bioreactor culture is three replicates for the first 75 hours and two replicates for the 75-120 hours.<br>For plant leaf STOX expression and infiltration, the sample size is three. According to previous literatures, for heterologous expression in Nicotiana benthamiana leaves, 3 replicates (one leaf for each replicate) were typically used for each biosynthetic gene being tested.<br>No statistical methods were used to predetermine sample size.
Data exclusions	No data exclusions.
Replication	Data are obtained from all replicates and all attempts are successful.
Randomization	In plant tests, three replicates were randomized among different plants such that no plant contained multiple replicates from the same condition being queried. No randomization on grouping was performed in the yeast culture experiments, since all replicates are processed to be test or measured in this study and they share the same genotype and growth condition. During LC-MS analysis, samples were randomized for loading order.
Blinding	Blinding was not performed in the study. Because the experiments did not involve any animals or human participants. For yeast cell culturing, seed cells are cultured from single colonies and then inoculated into different fermentation conditions described in the manuscript. The results are objective.

Reporting for specific materials, systems and methods

We require information from authors about some types of materials, experimental systems and methods used in many studies. Here, indicate whether each material, system or method listed is relevant to your study. If you are not sure if a list item applies to your research, read the appropriate section before selecting a response.

Materials & experimental systems

n/a	Involved in the study	
☒	☐	Antibodies
☒	☐	Eukaryotic cell lines
☒	☐	Palaeontology and archaeology
☒	☐	Animals and other organisms
☒	☐	Clinical data
☒	☐	Dual use research of concern

Methods

n/a	Involved in the study	
☒	☐	ChIP-seq
☒	☐	Flow cytometry
☒	☐	MRI-based neuroimaging
